# Analysis of biodegradation performance of furfural and 5-hydroxymethylfurfural by *Amorphotheca resinae* ZN1

**DOI:** 10.1186/1754-6834-7-51

**Published:** 2014-04-05

**Authors:** Hong Ran, Jian Zhang, Qiuqiang Gao, Zhanglin Lin, Jie Bao

**Affiliations:** 1 State Key Laboratory of Bioreactor Engineering, East China University of Science and Technology, 130 Meilong Road, Shanghai 200237, China; 2 Department of Chemical Engineering, Tsinghua University, Beijing 100084, China

**Keywords:** Biodegradation, furfural, 5-hydroxymethylfurfural, *Amorphotheca resinae* ZN1, lignocellulose, pretreatment, oxygen supply, substrate priority

## Abstract

**Background:**

Furfural and 5-hydroxymethylfurfural (HMF) are the degradation products of lignocellulose during pretreatment operations and significantly inhibit the consequent enzymatic hydrolysis and fermentation processes. The biodetoxification fungus *Amorphotheca resinae* ZN1 had demonstrated its excellent capacity on degrading lignocellulose derived inhibitors and helped the fermentation processes to achieve high yield of ethanol and biochemicals. Analysis of the biological degradation performance of furfural and HMF by *A. resinae* ZN1 will provide essential information for their fast and complete removal from the pretreated lignocellulose materials and facilitate the consequent ethanol fermentation.

**Results:**

The degradation performance of furfural and HMF by *A. resinae* ZN1 was investigated by capturing intermediate metabolic products at various culture conditions. *A. resinae* ZN1 converts furfural/HMF into furfuryl/HMF alcohols and furoic/HMF acids simultaneously at aerobic condition, and only the corresponding furfuryl/HMF alcohols are obtained at anaerobic condition. The existence of glucose accelerates the degradation rate of furfural and HMF by *A. resinae* ZN1 and the cell mass growth rate aerobically. Remarkably, glucose is not consumed before furfural or HMF is degraded to a low threshold concentration. The finding suggests that furfural or HMF has a substrate priority of utilization by *A. resinae* ZN1 than glucose. This property may help the detoxification of furfural and HMF to be operated without consuming glucose.

**Conclusions:**

The biological degradation performance of furfural and HMF by *A. resinae* ZN1 was investigated experimentally. Oxygen supply is important on the complete biodegradation of furfural and HMF by *A. resinae* ZN1. Furfural or HMF has the priority of substrate utilization than glucose by *A. resinae* ZN1. This study provided important information for detoxification enhancement and strain modification.

## Background

Pretreatment is the crucial step to overcome the biorecalcitrance of lignocellulose for its hydrolysis and fermentation into biofuels and biochemicals [1,2]. Currently, all the available pretreatment methods, including the classical dilute acid, steam explosion [3], and alkali [4], as well as the new concepts using ionic liquids [5] and microwave [6], are inevitable to convert partial lignocellulose biomass into various small compounds, including furan derivatives such as furfural and 5-hydromethylfurfural (HMF), organic acids such as acetic acid, formic acid, and levulinic acid, as well as phenolic compounds such as vanillin, syringaldehyde, 4-hydroxybenzaldehyde, coniferyl aldehyde, ferulic acid, and cinnamic acid [7,8]. These compounds are strong inhibitors of cellulase enzyme and fermenting strains [9,10]. Among the inhibitor compounds that were mentioned, furfural and HMF are considered as the major inhibitors because the two are high in concentration and strong in inhibition strength to ethanol fermenting strains [10,11]. Furfural or HMF attacks the cell membrane and interferes with intracellular metabolism [12]. Once it has entered cells, its hydrophobic groups combine with various intracellular enzymes, making them lose the ability to combine with the substrate, especially glycolysis enzymes [13,14].

Although a mild pretreatment may yield fewer inhibitor compounds, the practical industrial processes still need the intensively pretreated lignocellulose feedstock for a high bioconversion yield. The intensive pretreatment inevitably accompanies the high inhibitor-generation, thus, the complete removal of inhibitors from pretreated lignocellulose materials using physical, chemical, or biological methods, or *detoxification*, is an important step [15]. The common methods include water washing, over-liming, ammonia neutralization, ion exchange absorption, solvent extraction and activated charcoal treatment [16-18]. However, these methods consume massive amounts of fresh water and generate a great amount of polluting waste water, along with significant loss of fine lignocellulose particles and fermentative sugars [19]. In recent years, a new method, biodetoxification, was proposed using unique microorganisms to degrade the inhibitor compounds biologically [20,21]. Biodetoxification had been applied to various biorefining processes and has demonstrated its unique advantages, such as mild condition, complete removal of inhibitors, low sugar-loss, and minimum waste-water generation [20-22].

Several biodetoxification strains had been applied to degrade the lignocelluloses-derived inhibitor compounds. Zhang *et al*. [23] found that *Clostridium acetobutylicum* ATCC 824 converts furfural/HMF to furfuryl alcohol/2, 5-bis-hydroxymethylfuran. Koopman *et al*. [24] introduced the furfural/HMF oxidoreductase gene hmfH into *Pseudomonas putida* S12 for conversion of highly concentrated HMF (6.3 g/L) to its low inhibitory mesostate, 2, 5-furandicarboxylic acid. Liu *et al*. [10] studied the stress tolerance of *Saccharomyces cerevisiae* ATCC 211239 and NRRL Y-12632, as well as *Pichia stipitis* NRRL Y-7124 on furfural and HMF, and the results showed that *S. cerevisiae* NRRL Y-12632 transformed furfural/HMF into furfuryl alcohol/2,5-bis-hydroxymethylfuran. Nichols *et al*. [25] isolated a fungal, *Coniochaeta ligniaria* NRRL30616, which could convert furfural to both furfuryl alcohol and furoic acid. Taherzadeh *et al*. [26] studied *S. cerevisiae* CBS 8066 and discovered that it converted HMF into C_4_H_3_O-CO-COH (COOH)-CH_3_, then became HMF alcohol. Liu *et al*. [27] analyzed the evolutionarily engineered *S. cerevisiae* NRRL Y-50049 strain and found that a well-maintained redox balance is crucially important for the robust tolerance of the yeast to furfural and HMF when they transformed furfural/HMF into furfuryl alcohol/HMF alcohol.

In our previous study, a kerosene fungus strain *Amorphotheca resinae* ZN1 was isolated from microbial communities on pretreated corn stover materials. *A. resinae* ZN1 was found to quickly degrade various furan derivatives, organic acids, and phenolic compounds. Then it was practically applied to degrade the inhibitors on dilute acid-pretreated corn stover and the consequent simultaneous saccharification and fermentation for production of ethanol, microbial lipid, and lactic acid, with dramatic decreases in fresh waste use, waste water generation, solids loss and energy consumption [21]. This work promoted *A. resinae* from being considered a harmful strain (that grows in aviation kerosene fuel tanks and blocks pipelines) to a new field, since its discovery in the 1970s [28].

In this study, the degradation pathways of the two furan derivatives from the pretreated lignocellulose, furfural and HMF, by *A. resinae* ZN1, were experimentally investigated and analyzed. First, the degradation products from furfural and HMF were identified when furfural or HMF was used as the sole carbon source at different oxygen levels. Then, the effect of the presence of glucose on the degradation and its pathways of *A. resinae* ZN1 were investigated. Finally, the degradation pathways of furfural and HMF by *A. resinae* ZN1 were proposed based on the experimental results and the similar previous pathways studies [29]. This study provided essential information for enhanced understanding of the degradation pathway of *A. resinae* ZN1 for the future improvement of detoxification efficiency and metabolic modification of the strain.

## Results and discussion

### Degradation of furfural and HMF when furfural or HMF was the sole carbon source

The metabolic performance of *A. resinae* ZN1 using furfural or HMF as the sole carbon source (without glucose addition in the culture medium) was tested under aerobic and anaerobic conditions. Before these biodegradation experiments of furfural and HMF, the control experiments of furfural and HMF degradation in the bioreactor were carried out in the aerobic condition but in the absence of microbes (Additional file 1: Figure S1). When no microbe existed in the culture system, the aerated airflow blew approximately 50% volatile furfural out within 50 hours, but no furfuryl alcohol or furoic acid was observed (Additional file 1: Figure S1a). On the other hand, furfuryl alcohol concentration remained constant within the 120-hour experimental period, and no furfural or furoic acid formation was observed (Additional file 1: Figure S1b). For HMF, both HMF and HMF alcohol remained constant in the 120-hour period, and no HMF acid formation was observed (Additional file 1: Figure S1c-d). The results indicate that the aeration did not lead to the direct oxidation of either furfural/furfuryl alcohol, or HMF/HMF alcohol into their corresponding acid forms. This is in agreement with the previous studies showing that furfural could not be oxidized by air directly at ambient temperature [30-33].

Furfural was degraded slowly in the strictly anaerobic culture of *A. resinae* ZN1 with furfural as the sole carbon source (Additional file 1: Figure S2a). Furfuryl alcohol increased gradually from the very beginning and minor furoic acid was observed at the late stage of the culture. HMF was degraded in a similar way with furfural in the anaerobic condition, but with a faster rate (Additional file 1: Figure S2b). The HMF alcohol increased correspondingly with the decrease of HMF, but no HMF acid was observed during the HMF degradation.

**Figure 1:**
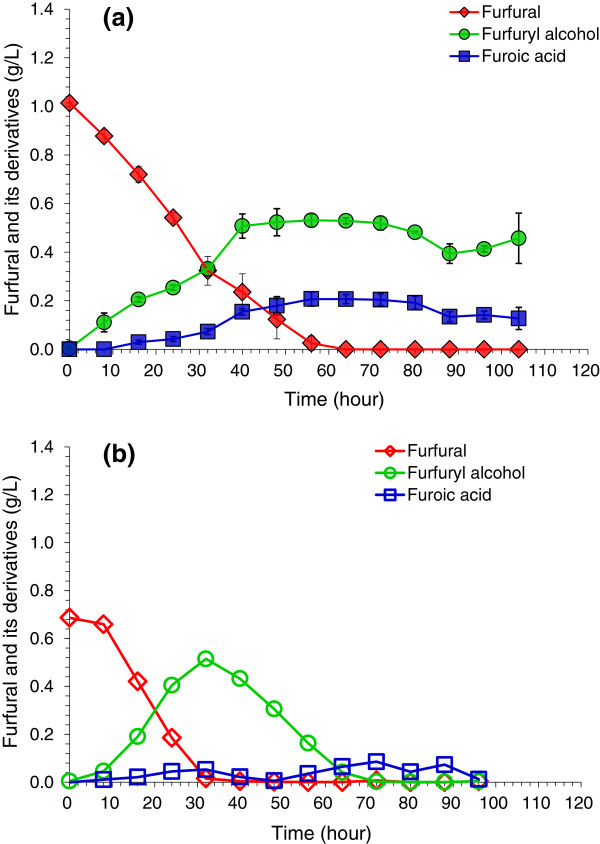
**Degradation of furfural by *A. resinae *ZN1 in the aerobic condition without glucose. (a)** Air rate at 0.625 volume per volume per minute (vvm); **(b)** Air rate at 1.25 vvm. Conditions: inoculum 20% (v/v), 28°C, pH 5.5, 100 rpm.

Figure 1 shows the time courses of the degradation of furfural in the aerobic condition with a different air rate when furfural was used as the sole carbon source. At the aeration rate of 0.625 volume per volume per minute (vvm) in Figure 1a, the sum of furfuryl alcohol and furoic acid was approximately 7.5 mmol after 50 hours of cultivation. The data accounted for 75% of the original furfural (10 mmol) approximately, indicating that approximately 25% of the original furfural was lost by the aeration at 0.625 vvm. At the aeration rate of 1.25 vvm in Figure 1b, the sum of furfuryl alcohol and furoic acid was approximately 5.8 mmol after 30 hours, accounting 80% of the original furfural (7.2 mmol), thus, approximately 20% of furfural was lost. The loss of furfural in the presence of microbes was only less than half compared to the loss of furfural at the same air rate in the control experiment, perhaps due to the simultaneous removal of furfural by aeration and biodegradation, thus, the furfural concentration more reduced quickly than that in the control experiment. Figure 1 indicates that the degradation rate of furfural was improved significantly in the aerobic condition compared to that in the anaerobic condition when furfural was used as sole carbon source, even considering the aeration loss of furfural. Furfural was completely degraded after 64 hours, compared to only 35% degradation of furfural in the anaerobic condition over the same time period. The quick formation of furfuryl alcohol and furoic acid was also observed with the decreasing furfural. Figure 1b shows the rates were similar for furfural degradation and furfuryl alcohol formation, and decreased furoic acid level at the two air input rates (0.625 vvm and 1.25 vvm). The major difference with the increased air input was the faster decrease of furfuryl alcohol when it reached the maximum.

**Figure 2:**
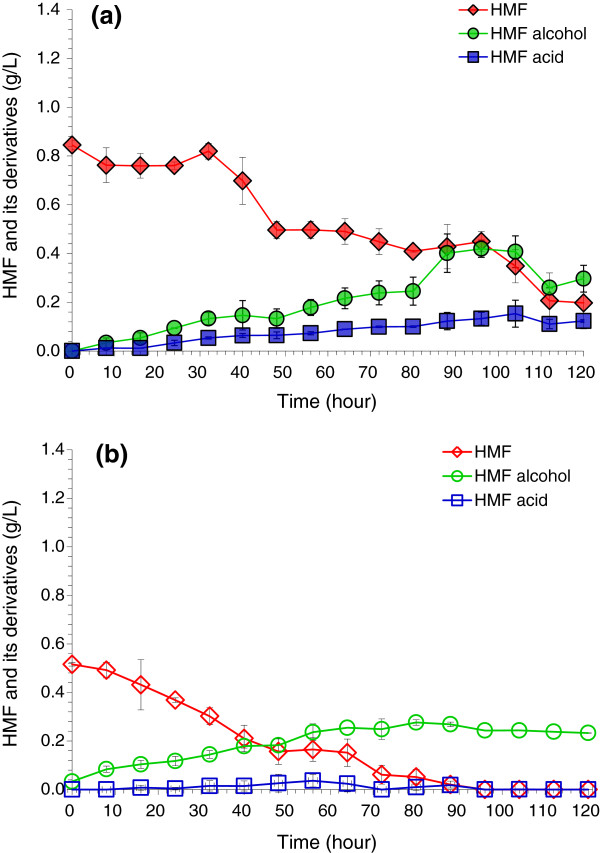
**Degradation of 5-hydroxymethylfurfural (HMF) by *A. resinae *ZN1 in the aerobic condition without glucose. (a)** Air rate at 0.625 volume per volume per minute (vvm); **(b)** Air rate at 1.25 vvm. Conditions: inoculum 20% (v/v), 28°C, pH 5.5, 100 rpm.

Figure 2 shows the time courses of HMF degradation in different aerobic conditions (0.625 vvm and 1.25 vvm) when HMF was used as the sole carbon source. The HMF decrease was approximately balanced with the increase in HMF alcohol and HMF acid in both aeration rates. This result was in agreement with the control experiment for HMF (Additional file 1: Figure S1c-d) in which both HMF and HMF alcohol remained constant during aeration. Compared to the strictly anaerobic degradation of HMF (Additional file 1: Figure S2b, the HMF degradation at the two aeration rates (0.625 vvm and 1.25 vvm) was not significantly changed. The major observable change was the formation of HMF acid with the aerobic culture, whereas almost no HMF acid was observed in the anaerobic culture (Additional file 1: Figure S2b).

A significant increase of cell mass was observed with the aerobic culture compared to the anaerobic culture. However, the change in cell mass was not qualitatively recorded during the degradation culture, because of difficulties in sampling due to severe flocculation of the cell mycelium on the stirrer and the wall of the fermentor.

These results suggest that *A. resinae* ZN1 degrades furfural and HMF into their less toxic metabolites, furfuryl alcohol and HMF alcohol, respectively, without other carbon sources in both anaerobic and aerobic conditions. With the aerobic conditions, furfural and HMF were converted into the corresponding alcohols, as well as their corresponding acids. However, with the anaerobic condition, furfural and HMF were converted into alcohols only with the further conversion to the corresponding acids. In respect to the degradation rate, furfural degradation was significantly accelerated in the aerobic condition compared to the rate with the anaerobic condition. However, HMF degradation was not significantly affected by the switch of anaerobic to the aerobic culture, indicating that the HMF degradation was not sensitive to the presence of oxygen in its degradation pathways.

### Degradation of furfural and HMF when glucose was present with furfural or HMF

**Figure 3:**
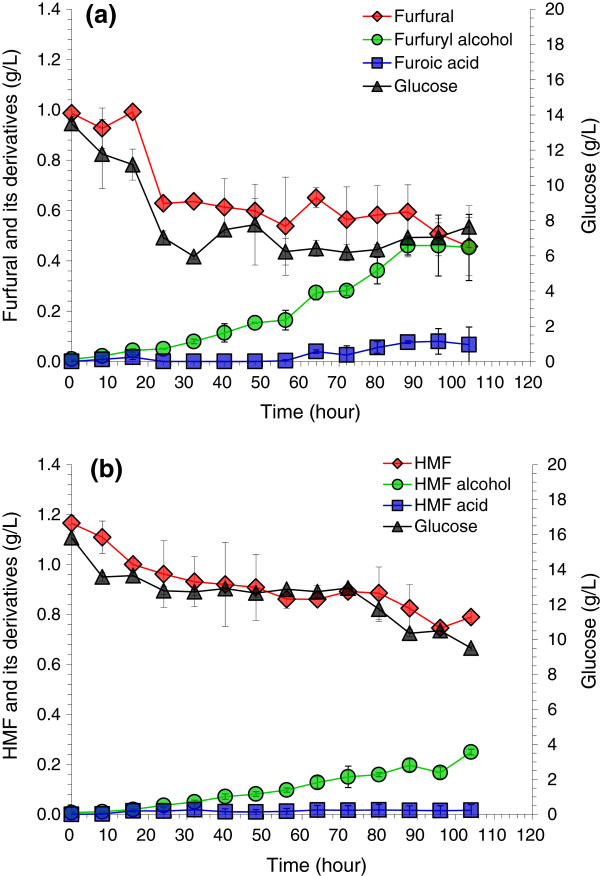
**Degradation of furfural and 5-hydroxymethylfurfural (HMF) by *A. resinae *ZN1 in anaerobic conditions with glucose. (a)** furfural; **(b)** HMF. Conditions: inoculum 20% (v/v), 28°C, pH 5.5, 100 rpm.

Pretreatment is generally accompanied with the partial hydrolysis of cellulose into glucose, thus, biodetoxification is frequently operated in the presence of a certain level of glucose. Therefore, the degradation of furfural and HMF by *A. resinae* ZN1 in the presence of glucose was examined. First, the furfural and HMF degradation in the presence of glucose were investigated with the strictly anaerobic condition as shown in Figure 3. The result indicates that both the degradation of furfural and HMF, as well as the formation of furfuryl alcohol or HMF alcohol was very slow compared to that without the addition of glucose (Additional file 1: Figure S2). Glucose consumption was also very slow. Only half of the initial glucose and inhibitors were degraded (furfural, Figure 3a), or less than half were degraded (HMF, Figure 3b) in the overall degradation culture period of 104 hours). The results revealed that the presence of glucose inhibited, rather than promoted, the degradation metabolism of furfural and HMF in the anaerobic condition.

**Figure 4:**
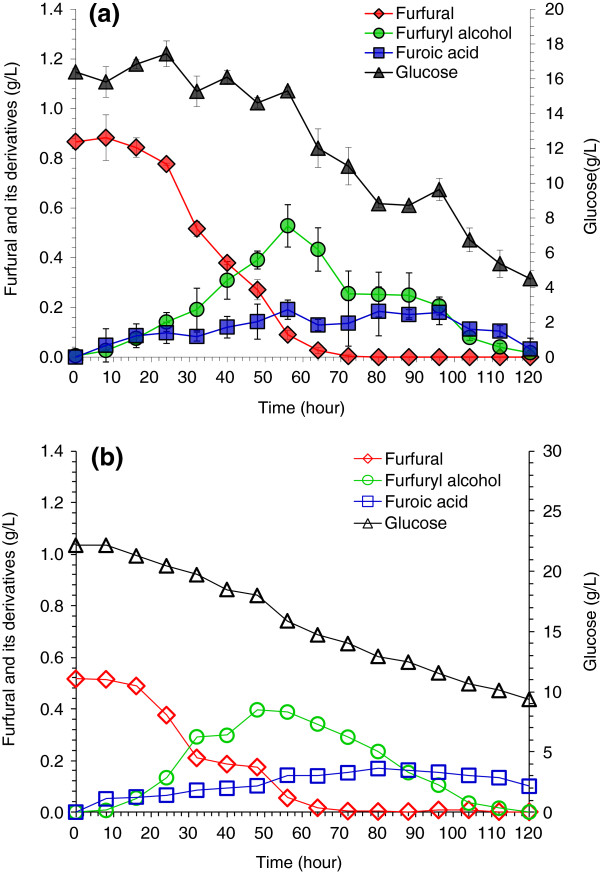
**Degradation of furfural by *A. resinae *ZN1 in aerobic conditions with glucose. (a)** Air rate at 0.625 volume per volume per minute (vvm); **(b)** Air rate at 1.25 vvm. Conditions: inoculum 20% (v/v), 28°C, pH 5.5, 100 rpm.

However, in the aerobic condition, the degradation of furfural and HMF in the presence of glucose was accelerated significantly. Figure 4 shows that furfural was completely degraded within 60 to 70 hours at the two aeration rates, and the degradation rates at 0.625 vvm (Figure 4a) and 1.25 vvm (Figure 4b) were approximately the same. Also the furfural decrease at 56 hours was approximately balanced with the increase of furfuryl alcohol and furoic acid, in which there was only 6% loss of furfural by aeration at 0.625 vvm (Figure 4a), and was almost completely balanced at 1.25 vvm (Figure 4b). Figure 4 indicates that furfuryl alcohol was formed with the decreasing furfural, then declined from its maximum, and the formation of furoic acid was observed.

**Figure 5:**
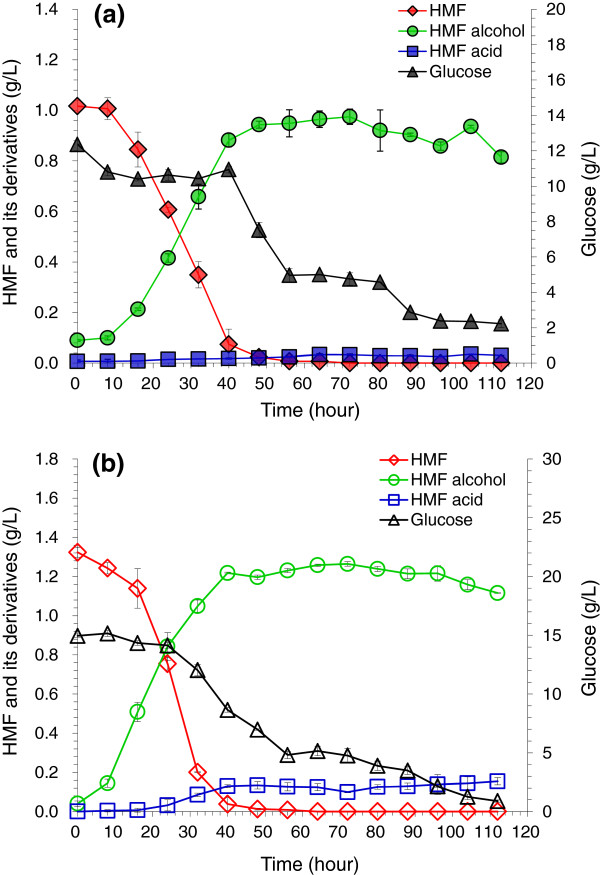
**Degradation of 5-hydroxymethylfurfural (HMF) by *A. resinae *ZN1 in aerobic conditions with glucose. (a)** Air rate at 0.625 volume per volume per minute (vvm); **(b)** Air rate at 1.25 vvm. Conditions: inoculum 20% (v/v), 28°C, pH 5.5, 100 rpm.

Figure 5 shows that HMF was completely degraded within 60 hours at the two aeration rates, and similarly to furfural degradation in Figure 4, the increase of aeration rate did not give observable effects on HMF degradation. The balance in mass with the HMF decrease was in agreement with the formation of HMF alcohol and HMF acid, and similar to furfural degradation Figure 5 indicates that HMF alcohol was produced correspondingly with decreasing HMF, but HMF alcohol was not reduced from the maximum, and only a small amount of HMF acid was formed.

Figures 4 and 5 reveal an interesting phenomenon where by in the aerobic condition glucose consumption was very slow at the beginning of the culture, but was suddenly accelerated when furfural or HMF was almost completely degraded (more than 90% of furfural or HMF was degraded). The finding suggests that furfural and HMF substrates had the priority for *A. resinae* ZN1 utilization rather than glucose in the aerobic condition. This important property of *A. resinae* ZN1 may help the detoxification of furfural and HMF without consuming glucose, thus, glucose could be preserved in the detoxification step and utilized in the consequent fermentation step for production of ethanol.

In the degradation experiment, the cell mass was not quantitatively measured because the hyphae and spores constantly aggregated and adhered to the wall and the stirrer of the fermentor. *A. resinae* ZN1 grew in the aerobic condition, but almost ceased growth in anaerobic conditions. When glucose was added to the medium, *A. resinae* ZN1 remained as spores with minor hyphae in the early period of culture, and then the spores turned black because of the generation of melanins, dark brown or black pigments located in cell walls [34,35]. Finally, a thick spore layer was absorbed onto the fermentor wall and a large amount of hyphae formed inside the culture medium.

### Analysis of degradation pathways of furfural and HMF by *A. resinae* ZN1

Biological degradation of furfural and HMF is an interesting topic and various pathways for different microorganisms have been proposed [29,36]. In this study, the degradation pathway for furfural and HMF by *A. resinae* ZN1 was carefully investigated for its importance in inhibitor removal in lignocellulose biorefinery processing. Some interesting results are summarized as follows: 1) *A. resinae* ZN1 converts furfural into furfuryl alcohol as the first step of degradation in all the experimental conditions. Under the aerobic condition (sufficient oxygen supply), *A. resinae* ZN1 converts furfural into furfuryl alcohol and furoic acid; under the strictly anaerobic condition (without oxygen supply), the conversion of furfural stops at furfuryl alcohol, furoic acid is not generated; 2) similar to furfural conversion, in all the experimental conditions *A. resinae* ZN1 converts HMF into HMF alcohol first. In the aerobic condition, *A. resinae* ZN1 converts HMF into HMF alcohol and HMF acid in the absence of glucose, but stops at the HMF alcohol step, and almost no HMF acid is generated without glucose; under anaerobic condition, similar to furfural degradation, the conversion of HMF stops at the HMF alcohol; and 3) *A. resinae* ZN1 has obvious priority over glucose for the utilization of furfural and HMF. When furfural or HMF is maintained at high levels, glucose is almost un-utilizedby *A. resinae* ZN1. The quick uptake of glucose by *A. resinae* ZN1 occurs only when furfural or HMF is reduced to a low-threshold concentration (lower than 0.2 g/L).

**Figure 6:**
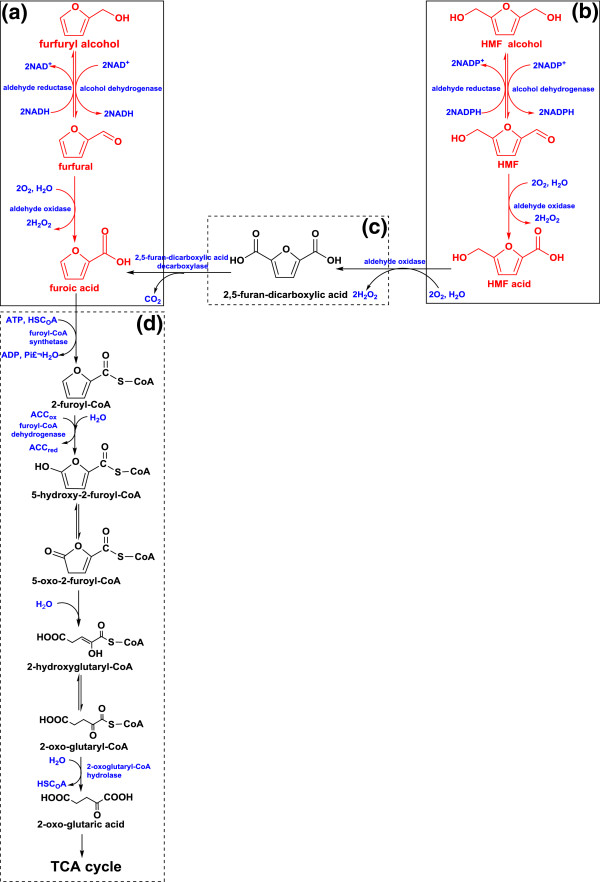
**The degradation pathway for furfural and 5-hydroxymethylfurfural (HMF) by *A. resinae *ZN1.** Solid boxes **(a and b)**, the metabolic pathway were speculated on from experimental phenomena (**a**, furfural; **b**, HMF). Dotted boxes **(c and d)**, blue (enzymes), green (substrates), and red (compounds) were speculated on based on the existing literature and chemical reactions. NADH, nicotinamide adenine dinucleotide; NADPH, nicotinamide adenine dinucleotide phosphate; NADP^+^, oxidized form of dicotinamide adenine dinucleotide phosphate; NADH, reduced form of nicotinamide-adenine dinucleotid; TCA, tricarboxcylic acid.

Based on the above experimental results for *A. resinae* ZN1 and the previous pathway studies of *Cupriavidus basilensis* HMF14 by Koopman *et al*. [29], the degradation pathway of *A. resinae* ZN1 for furfural and HMF could be proposed as shown in Figure 6.

The proposed pathway is as follows: 1) Furfural or HMF is converted to the corresponding furfuryl alcohol or HMF alcohol, as the first step by alcohol dehydrogenase or aldehyde reductase, instead of the conversion to furoic acid or HMF acid directly (boxes A and B in Figure 6). The reason is that forboth furfural and HMF, furoic acid and HMF acid has the stronger toxicity to microbes than furfuryl alcohol and HMF alcohol [7], thus the first step of conversion releases *A. resinae* ZN1 from the most toxic inhibitor of furfural or HMF, to the relatively weaker inhibitors of furfuryl alcohol or HMF alcohol. Also, the existence of furfural (HMF) is more sensitive to the activity of aldehyde oxidase than to alcohol dehydrogenase, thus, the conversion to furfuryl (HMF) alcohol is preferred to furoic (HMF) acid [37]; 2) under the aerobic condition, furfuryl alcohol is oxidized to furfural again by alcohol dehydrogenase or aldehyde reductase, and then to furoic acid, consequently by aldehyde oxidase in the presence of oxygen (boxes A and B in Figure 6). In the two consequent conversion steps, furfural or HMF is maintained at a very low level so that it does not affect the microbial growth and metabolism; 3) under anaerobic conditions, the degradation pathway of furfural or HMF stops at the step of furfuryl alcohol or HMF alcohol and the conversion into the corresponding acids does not occur. The presence of oxygen is the prerequisite condition for the degradation of furfuryl alcohol or HMF alcohol to the corresponding acids. The oxygen-independent aldehyde oxidase may be missing or inactive for the conversion from furfural (HMF) to furoic (HMF) acid in the anaerobic condition; 4) based on analysis of *C. basilensis* HMF14 by Koopman *et al*., it is estimated that HMF acid is oxidized to 2,5-furan-dicarboxylic acid by aldehyde oxidase, then to furoic acid by 2,5-furan-dicarboxylic acid decarboxylase [29] as shown (Figure 6, box c). By these two step conversions, the HMF degradation pathway (Figure 6, box b) joins the furfural degradation pathway (Figure 6, box a) at the final step of furoic acid formation. Then furoic acid is converted to 2-oxo-glutaric acid through six steps, then joins the tricarboxylic acid (TCA) cycle to complete the degradation as shown (Figure 6, box D) [29]; 5) the reason for prior utilization of furfural and HMF compared to glucose might come from the inhibition of furfural and HMF to the central carbon metabolism. Unless furfural or HMF is reduced to a low-threshold concentration, glycolysis or the TCA cycle will not be initiated for glucose utilization and cell growth. This property helps the biological detoxification process to reserve the glucose sugar for the final fermentation of target products such as ethanol, instead of the consumption on degradation of inhibitor compounds; 6) degradation of furfural and HMF by *A. resinae* ZN1 may have a strong cofactor preference in its metabolic pathways [38,39]. The reduction of HMF is coupled with the oxidation of nicotinamide adenine dinucleotide phosphate (NADPH) [40], whereas NADPH is mainly produced by pentose phosphate pathway and its concentration level is highly associated with glucose [41,27]. Perhaps there is a similar mechanism; the existence of HMF in *A. resinae* ZN1 enhances the expression of the related genes, such as *ZWF1*, *GND1*, and *GND2* in glucose metabolism of yeast, and then the concentration of NADPH is elevated, thus, the degradation of HMF is increased [27]. On the other hand, the degradation of furfural is coupled with the oxidation of the reduced form of nicotinamide-adenine dinucleotid (NADH) [42], and the NADH level is highly associated with respiration (sufficient oxygen supply), with or without the presence of glucose.This explains why the presence of glucose affects the degradation rate of HMF significantly, but is less sensitive to furfural degradation.

## Conclusion

The degradation performance of furfural and HMF by the biodetoxification fungus *A. resinae* ZN1 was investigated and analyzed experimentally. Oxygen supply and glucose limitation were found to play crucial roles in the biodegradation of furfural and HMF. Under aerobic conditions, furfural and HMF were converted into the corresponding alcohols and acids at first, then further degraded. Under the anaerobic condition, furfural and HMF were converted into alcohols only without the further conversion to the corresponding acids. The threshold for the presence of glucose provides a possibility for retaining glucose for the subsequent fermentation steps for production of ethanol or other fermentation products. The results provide important information for process intensification of inhibitor removal from the pretreated materials and strain modifications for enhancing the capacity for degradation.

## Methods

### Strain and medium

*A. resinae* ZN1 was isolated in our previous work [21] and stored in the China General Microbiological Culture Collection Center (CGMCC), Beijing, China with the registration number CGMCC 7452. *A. resinae* ZN1 was stored and transferred on a potato dextrose agar medium (PDA) slant. The PDA medium was prepared by boiling 200 g of peeled and sliced potatoes in 1 L dH_2_O for 30 minutes before being filtered. We added 20 g glucose and 20 g agar to the filtrate and sterilized this at 121°C for 20 minutes, then stored it at 4°C.

The culture media of *A. resinae* ZN1 included: 1) carbon source medium: KH_2_PO_4_ 2 g/L, (NH_4_)_2_SO_4_ 1 g/L, MgSO_4_ 7H_2_O 1 g/L, CaCl_2_ 0.5 g/L, yeast extract 1 g/L, glucose 20 g/L and 2) inorganic salt medium: KH_2_PO_4_ 2 g/L, (NH_4_)_2_SO_4_ 1 g/L, MgSO_4_ 7H_2_O 1 g/L, CaCl_2_ 0.5 g/L.

### Inhibitor degradation cultures

*A. resinae* ZN1 seeds were cultivated in Erlenmeyer flasks and the subsequent bioconversion experiments were performed in a 3-L fermentor (Baoxing Biotech Co., Shanghai, China). Spores of *A. resinae* ZN1 were washed from two PDA slants to prepare 20 ml of spore suspension, then were inoculated into 200 ml of seed medium into a 500-ml flask at a 10% (v/v) inoculation ratio. The culture was incubated for two days at 28°C when glucose was used as the carbon source, or four days when furfural or HMF was used as the carbon source. The cell mycelium was harvested and washed by 250 ml sterile deionized water twice to remove the remaining glucose, then suspended in 200 ml of inorganic salt medium as the seed culture.

The experiments of furfural and HMF degradation were carried out in the same 3-L fermentor containing 1 L of fermentation medium or inorganic salt medium with 1.0 g/L of furfural or HMF. The fermentation was inoculated by the seed culture at 20% (v/v) and cultured at 28°C and 100 rpm for approximately one week until the furfural or HMF added was almost completely degraded. The dissolved oxygen was maintained at a constant concentration by regulating the amount of air entering through an air sparger. The anaerobic culture was maintained by sparging nitrogen for 30 minutes after inoculation. The required pH value was maintained by the addition of 2 M HCl or 2 M NaOH. 1 ml of the biotransformation mixtures were centrifuged (15,800 × g, 6 minutes) to remove the cell mycelia, and were then stored at 4°C until use.

### Analytical methods

Furan and its derivatives, 2-furaldehyde (furfural), furfuryl alcohol, 2-furoic acid (furoic acid), 5-hydroxymethylfurfural (HMF), 5-hydroxymethylfurfuryl alcohol (HMF alcohol), and 5-hydroxymethylfuroic acid (HMF acid) were analyzed using reverse-phase HPLC (LC-20AT, Japan), equipped with a YMC-Pack ODS-A column (YMC, Tokyo, Japan) and an SPD-20A UV detector (Shimadzu, Kyoto, Japan). Furfural, furfuryl alcohol, and furoic acid were analyzed using 50% acetonitrile solution as the mobile phase at 1.0 ml/minute at the column temperature of 35°C and the detection wavelength of 220 nm. HMF, HMF alcohol, and HMF acid were analyzed using the following gradient: the initial flow phase was composed by pure water (pump A) and acetonitrile (pump B) at a ratio of 95% to 5%; first, acetonitrile was increased from 5% to 100% over 0 to 15 minutes; then, acetonitrile was decreased from 100% to 5% over 15 to 20 minutes; finally, acetonitrile was used at 5% over 20 to 30 minutes. The flow rate was 0.6 ml/minute, the column temperature was 35°C, and the detector wavelength was 230 nm [29,43].

Glucose was analyzed using HPLC (LC-20 AD, refractive index detector RID-10A, Shimadzu, Kyoto, Japan) with a Bio-rad Aminex HPX-87H column at the temperature of 65°C. The mobile phase was 5 mM H_2_SO_4_ at the rate of 0.6 ml/minute. All samples were centrifuged at 15,800 × g for 6 minutes, and then filtered through a 0.22-μm filter before analysis.

## Abbreviations

A. resinae ZN1: *Amorphotheca resinae* ZN1; CGMCC: China General Microbiological Culture Collection Center; HMF: 5-hydroxymethylfurfural; HMF alcohol: 5-hydroxymethylfurfuryl alcohol; HMF acid: 5-hydroxymethylfuroic acid; HPLC: high performance liquid chromatography; NADPH: nicotinamide adenine dinucleotide phosphate; NADP+: Oxidized form of dicotinamide adenine dinucleotide phosphate; NADH: reduced form of nicotinamide-adenine dinucleotide; PDA: potato dextrose agar; TCA: tricarboxylic acid cycle; Vvm: volume per volume per minute.

## Competing interests

The authors declare that they have no competing interests.

## Authors’ contributions

JB and HR designed the experiment; HR conducted the degradation experiment; JZ developed the *A. resinae* ZN1 strain culture procedure; QQG analyzed the metabolite identification method; ZLL analyzed the inhibitor degradation pathway; JB conceived the study; JZ, QQG, ZLL revised the manuscript critically; HR and JB wrote the manuscript. All authors read and approved the final manuscript.

## Supplementary Material

Additional file 1 **Control and anaerobic degradation experiments of furfural and 5-hydroxymethylfurfural (HMF). Figure S1.** Control experiments on furfural and HMF degradation under absence of microbes. Conditions: inorganic salt medium, air rate 0.625 volume per volume per minute (vvm), 28°C, pH 5.5, 100 rpm. **Figure S2.** Degradation of furfural and HMF by *A. resinae* ZN1 at anaerobic condition without glucose. **(a)** Furfural; **(b)** HMF. Conditions: inoculum ratio 20% (v/v), 28°C, pH 5.5, 100 rpm. Click here for file
