# Supplementary material for: Analysis of biodegradation performance of furfural and 5-hydroxymethylfurfural by Amorphotheca resinae ZN1
Source: Biotechnol Biofuels. 2014 Apr 5;7:51. doi: 10.1186/1754-6834-7-51 (PMC4101820; doi:10.1186/1754-6834-7-51)
Supplement: Additional file 1 — Control and anaerobic degradation experiments of furfural and 5-hydroxymethylfurfural (HMF). Figure S1. Control experiments on furfural and HMF degradation under absence of microbes. Conditions: inorganic salt medium, air rate 0.625 volume per volume per minute (vvm), 28°C, pH 5.5, 100 rpm. Figure S2. Degradation of furfural and HMF by A. resinae ZN1 at anaerobic condition without glucose. (a) Furfural; (b) HMF. Conditions: inoculum ratio 20% (v/v), 28°C, pH 5.5, 100 rpm. [file 1754-6834-7-51-S1.doc]

**Additional file 1: Control and** **anaerobic degradation experiments of furfural and HMF**

**Figure S1**. Control experiments on furfural and HMF degradation under no microbe existence. Conditions: inorganic salt medium, air rate at 0.625 vvm, 28 ^o^C, pH 5.5, 100 rpm.

**Figure S2**. Degradation of furfural and HMF by *A. resinae* ZN1 at anaerobic condition without glucose. (a) furfural; (b) HMF. Conditions: inoculum ratio 20% (v/v), 28 ^o^C, pH 5.5, 100 rpm.
